# Recovery of hypopituitarism in macroprolactinomas: a comparison of medical vs. surgical treatment. Results from a European multicenter study

**DOI:** 10.1007/s40618-025-02559-8

**Published:** 2025-03-04

**Authors:** Mario Detomas, Barbara Altieri, Isabella Nasi-Kordhishti, Alice Ryba, Linus Haberbosch, Francesco Chierigo, Timo Deutschbein, Martin Fassnacht, Pietro Mortini, Joerg Flitsch, Juergen Honegger, Marco Losa

**Affiliations:** 1 Department of Internal Medicine I, Division of Endocrinology and Diabetes, University Hospital, University of Würzburg, Würzburg, Germany; 2 Department of Endocrinology, William Harvey Research Institute, Barts and the London School of Medicine, Queen Mary University of London, London, UK; 3 Department of Neurosurgery, University Hospital Tübingen, , Tübingen, Germany; 4 Department of Neurosurgery, Medical Center Hamburg-Eppendorf, Hamburg, Germany; 5 Department of Endocrinology and Metabolism, European Reference Network on Rare Endocrine Diseases (ENDO-ERN), Charité Universitätsmedizin, Berlin, Germany; 6 Department of Urology, ASST Santi Paolo e Carlo, Milan, Italy; 7 Medicover Oldenburg MVZ, Oldenburg, Germany; 8 Department of Neurosurgery, IRCCS San Raffaele Scientific Institute, Vita-Salute University, Milan, Italy

**Keywords:** Prolactinoma, Hypogonadism, Adrenal insufficiency, Hypothyroidism, ACTH deficiency, TSH deficiency

## Abstract

**Context:**

Macroprolactinomas not only cause hypogonadism, but also other pituitary dysfunctions, like deficiency of adrenocorticotrophic hormone (ACTH) and thyroid-stimulating hormone (TSH). While dopamine agonist treatment shows varying recovery rates of these insufficiencies, surgical outcomes are less studied, and a direct comparison between treatments is lacking.

**Objective:**

To evaluate recovery of pituitary dysfunction in medically vs. surgically treated patients with macroprolactinoma.

**Design:**

Retrospective multicenter study including 104 patients with macroprolactinoma (44 surgically vs. 60 medically treated) with at least two hormonal deficiencies before treatment.

**Results:**

Before surgery, all patients presented with hypogonadotropic hypogonadism, 25 (57%) with ACTH-deficiency, and 32 (73%) with TSH-deficiency. 10 months post-surgery, prolactin normalized in 25 (57%) patients, while 19(43%), 15 (60%) and 10(31%) recovered from hypogonadism, ACTH-deficiency, and TSH-deficiency, respectively. Before medical therapy, hypogonadism was observed in all patients, ACTH-deficiency in 31 (52%), and TSH-deficiency in 50 (83%). After 12 months under dopamine agonists, prolactin levels normalized in 36 (60%) patients, 25(42%) recovered from hypogonadism, 17 (55%) from ACTH-deficiency, and 14(28%) from TSH-deficiency. No significant difference in recovery rates between surgical and medical treatment for hypogonadism (OR 1.633, *p* = 0.338), ACTH-deficiency (OR 0.462, *p* = 0.319), or TSH-deficiency (OR 0.584, *p* = 0.339) was observed. Large initial tumor size was a significant negative predictor of recovery for all hormone deficiencies (always *p* < 0.05), while prolactin normalization was a predictor of recovery of hypogonadism (*p* < 0.001).

**Conclusion:**

Both surgical and medical treatment allow for hormonal recovery in patients with macroprolactinoma, with no significant advantage for either approach. Initial tumor size and prolactin-normalization are predictors of recovery outcomes.

**Supplementary Information:**

The online version contains supplementary material available at 10.1007/s40618-025-02559-8.

## Introduction

Prolactinomas have an estimated prevalence of 37 cases per 100,000 people [1]. While 90% of prolactinomas are microadenomas (diameter < 1 cm), macroadenomas (≥ 1 cm) are less common but more prevalent in men than in women [1, 2].

Excessive prolactin secretion typically leads to hypogonadotropic hypogonadism in up to 90% of cases, irrespective of tumor size [2, 3]. However, tumor mass per se significantly increases the risk of hypogonadism and may also cause other anterior pituitary hormone insufficiencies. In detail, deficiencies of thyroid-stimulating hormone (TSH) and adrenocorticotrophic hormone (ACTH) have been reported in up to 50% of cases with macroprolactinomas [3–8]. Therefore, treatment should not only aim to normalize hyperprolactinemia and reduce tumor size but also restore normal pituitary function.

In patients treated with dopamine agonists, recovery rates for hypogonadism, ACTH deficiency, and TSH deficiency range from 44 to 83%, 17–64%, and 25–29%, respectively [3, 6, 7, 9]. On the other hand, hypopituitarism recovers in approximately one third of surgically treated patients [10, 11].

Due to the rarity of macroprolactinomas, the above reported studies are limited by small cohorts, and some have included male patients only. Moreover, although one study sought to analyze the recovery of hypopituitarism in patients with giant prolactinoma (macroprolactinoma > 4 cm) treated medically or surgically, it also included patients who had received radiotherapy, potentially introducing bias into the analysis [12].

This international multicenter study reports one of the largest cohorts of patients with macroprolactinoma and hypopituitarism and aims to compare the recovery of sex hormones, ACTH, and TSH deficiency after medical vs. surgical treatment in tertiary referral centers specialized in pituitary disorders [10, 13, 14].

## Subjects and methods

### Study design and population

Retrospective multicenter analysis of patients with macroprolactinomas treated in five tertiary reference centers for pituitary disorders between January 2000 and June 2024: Division of Endocrinology and Diabetes of the University Hospital Würzburg and Department of Endocrinology of the Charité University Hospital in Berlin (both centers included only patients with medical treatment); Department of Neurosurgery of the University Hospital San Raffaele of Milan (which included patients surgically and medically treated); Department of Neurosurgery of the University of Tübingen and Department of Neurosurgery of the University Hospital of Hamburg (both centers included only surgically treated patients). Of note, all three neurosurgical centers perform more than 100 pituitary transsphenoidal procedures each year [15]. The inclusion criteria for this study were: (i) adult patients; (ii) confirmed diagnosis of prolactinoma [1]; (iii) patients surgically treated or under dopamine agonists; (iv) reduction of prolactin levels after surgery or under medical therapy (as indicators of treatment effectiveness); (v) evidence of hypopituitarism at diagnosis, with at least 2 pituitary axes involved; (vi) follow-up of at least 6 months after surgery or initiation of medical treatment; (vii) patients undergoing surgery who previously treated with medical therapy for *≥* 3 months, and still had abnormally high prolactin levels and hypopituitarism. Patients who underwent radiotherapy before evaluation or with pituitary apoplexy were excluded from the analysis.

This multicenter study was conducted in accordance with the local ethical committees (approval numbers were 2023–300417-WF in Hamburg, NCH-02–21 in Milan, 578/2019 BO2 in Tübingen and 85/12 in Würzburg. According to the regulation of the “Ärztekammer-Berlin” 15.Abs.2, 2014, no approval of the local ethic committee of the University Hospital of Berlin (Charité) was needed. All research complied with the Declaration of Helsinki.

### Hormonal analysis

ACTH deficiency was diagnosed when morning serum cortisol levels (between 08:00–09:00 h) were low (< 5 µg/dL) and/or cortisol peaked below 18 µg/L after stimulation with synthetic ACTH (250 µg iv) [16]. Hypothyroidism was diagnosed when a subnormal serum free T4 (FT4) level (< 10 pmol/L) was associated with a low or low-normal TSH level (low levels according to local reference ranges) [17]. In postmenopausal women, hypogonadism was identified when serum LH and/or FSH levels were inappropriately low for their age (low levels according to local reference ranges). In premenopausal women, gonadotropin deficiency was diagnosed in the presence of amenorrhea or oligomenorrhea, when baseline gonadotropin levels were low or at the lower end of the normal range (low levels according to local reference ranges), along with persistently low estradiol levels (< 30 pg/mL) [17]. In men, hypogonadism was diagnosed when both testosterone and gonadotropin levels were below the normal age-specific reference ranges [18]. Unfortunately, an adequate diagnostic work-up for growth hormone deficiency (e.g. arginine-GHRH test), was available for only a few patients undergoing medical therapy and was not conducted at follow-up for any operated patients. Therefore, this analysis was not included in the study.

### Statistical analysis

Descriptive statistics included medians and interquartile ranges, as well as frequencies and proportions for continuous and categorical variables, respectively. The statistical significance of differences in medians and proportions was evaluated with the Wilcoxon and chi-square tests. Logistic regression models were fitted to test for differences in recovery of normal prolactin levels, hypogonadism, adrenal insufficiency, and hypothyroidism in patients treated with medical therapy vs. those treated with surgery.

For all statistical analyses, R software environment for statistical computing (version 3.4.3) and GraphPad Prism version 10 (GraphPad Software, San Diego, CA, USA) for graphics were used. All tests were two-sided with a level of significance set at *p* < 0.05.

## Results

### Patients undergoing surgery

We identified 44 patients with macroprolactinoma (27% women, median age 38 years) who underwent surgery and had at least two preoperative pituitary hormone deficiencies. The median prolactin level before surgery was 457 µg/L (IQR: 190–1322). Of note, 16 of the 44 patients (36%) were treated with dopamine agonists before surgery. Further patient-characteristics at the time of surgery are detailed in Table 1. Reasons for surgery were available in 38 (86%) of the patients: 15 patients (40%) chose surgery over medical therapy, 14 (37%) underwent surgery due to resistance to medical treatment, as determined by the endocrinologist and neurosurgeon, based on persistent hyperprolactinemia despite dose escalation of dopamine agonists and/or failure to achieve tumor shrinkage, 5 (13%) due to diagnostic uncertainty and 4 (10%) due to intolerance to medical treatment.

**Table 1:** Characteristics of patients at baseline

	Total (*n* = 104)	Surgery (*n* = 44)	Medical therapy (*n* = 60)	*p*-value
**Clinical characteristics**				
Women, n (%)	25 (24)	12 (27)	13 (22)	0.5
Age, years (IQR)	45 (32, 56)	38 (32, 48)	48 (34, 58)	0.07
**Tumor characteristics**				
Adenoma size, mm (IQR)	24 (14, 32)	22 (14, 30)	26 (19, 32)	0.2
Knosp grade				
0, n (%)	6 (6)	6 (14)	0 (0)	< 0.01
I, n (%)	26 (25)	12 (27)	14 (23)	
II, n (%)	21 (20)	11 (25)	10 (17)	
III, n (%)	15 (14)	6 (14)	9 (15)	
IV, n (%)	24 (23)	4 (9)	20 (33)	
Not available, n (%)	12 (12)	5 (11)	7 (12)	
**Biochemical characteristics**				
Prolactin, µg/l (IQR)	820 (315, 1820)	457 (190, 1322)	1220 (582, 2988)	< 0.001
Hypogonadotropic hypogonadism, n (%)	104 (100)	44 (100)	60 (100)	0.9
ACTH deficiency, n (%)	56 (54)	25 (57)	31 (52)	0.6
TSH deficiency, n (%)	82 (79)	32 (73)	50 (83)	0.2
**Prevalence of pituitary axis insufficiencies**				
2	71 (68)	31 (70)	40 (67)	0.7
3	33 (32)	13 (30)	20 (33)	

**Fig. 1:**
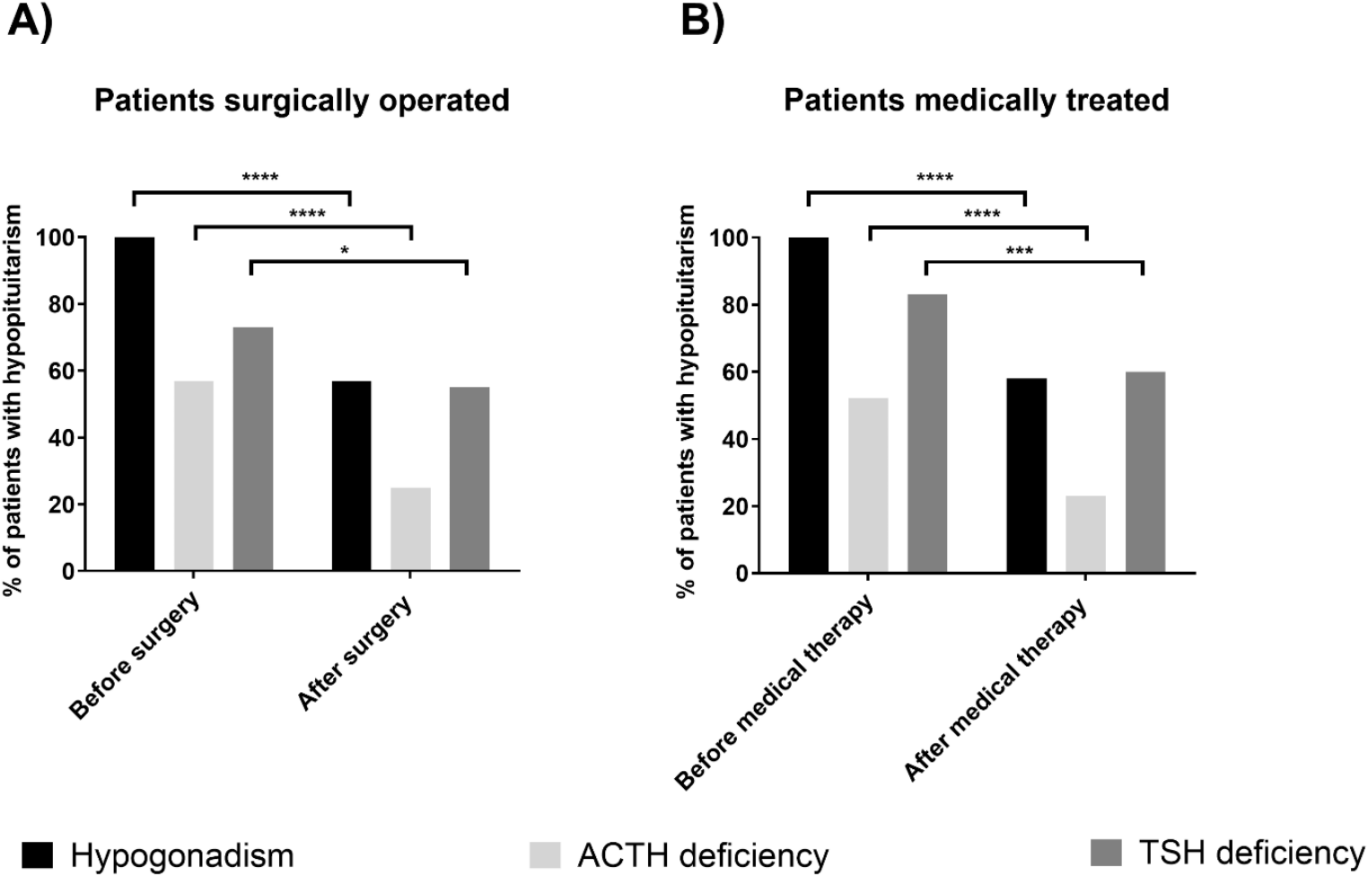
Prevalence of hypopituitarism at baseline and after treatment

In the context of the surgical intervention, all 44 patients presented with hypogonadotropic hypogonadism, while 25 (57%) had ACTH and 32 (73%) had TSH deficiency. After a median follow-up of 12 (IQR 6–12) months post-surgery, prolactin levels normalized in 28 (64%) patients. Hypogonadotropic hypogonadism persisted in 25 (57%) patients (Fig. 1), while 19 (43%) recovered. Postoperative ACTH deficiency was observed in 11 (25%) patients (Fig. 1), with one case who developed a new deficiency. Accordingly, 15 patients (60%) recovered from ACTH deficiency after surgery. TSH deficiency was present in 24 (55%) patients postoperatively (Fig. 1), including one newly affected case. Therefore, 10 patients (31%) showed recovery from TSH deficiency. Further data on hypopituitarism after surgery is summarized in Table 2.

**Table 2:** Rate of hypopituitarism after treatment

	Total (*n* = 104)	Surgery (*n* = 44)	Medical therapy (*n* = 60)	*p*-value
**Hormonal data after treatment**				
Prolactin normalization, n (%)	64 (62)	28 (64)	36 (60)	0.7
Hypogonadotropic hypogonadism, n (%)	60 (58)	25 (57)	35 (58)	0.9
ACTH deficiency, n (%)	25 (24)	11 (25)	14 (23)	0.8
TSH deficiency, n (%)	60 (58)	24 (55)	36 (60)	0.7
**Prevalence of pituitary axis insufficiencies**				
0	25 (24)	13 (30)	12 (20)	0.3
1	31 (30)	11 (25)	20 (33)	
2	29 (28)	11 (25)	18 (30)	
3	19 (18)	9 (20)	10 (17)	

### Patients receiving medical treatment

60 patients with macroprolactinoma (22% women, median age 48 years) and at least two pituitary hormone deficiencies who were treated with dopamine agonists were identified. Baseline characteristics of these patients are summarized in Table 1. The median prolactin level at baseline was 1220 µg/L (IQR: 582–2988). All 60 patients had hypogonadotropic hypogonadism, 31 (52%) ACTH deficiency, and 50 (83%) TSH deficiency. At baseline, no difference in terms of hypogonadism (100% vs. 100%), ACTH deficiency (50% vs. 60%, *p* = 0.155), and TSH deficiency (82% vs. 90%, *p* = 0.110) was observed in patients subsequently treated with cabergoline or bromocriptine. 50 (83%) patients were treated with cabergoline (median weekly dosage 1.0 mg) and 10 (17%) with bromocriptine (median weekly dosage of 26.3 mg).

After a median of 12 months (IQR 12–12) of medical treatment, prolactin levels normalized in 36 patients (60%), (each 60% under cabergoline and bromocriptine). Hypogonadotropic hypogonadism persisted in 35 (58%) patients (Fig. 1), while 25 (42%) patients recovered. Ongoing ACTH deficiency was observed in 14 (23%) patients (Fig. 1), facing 17 patients (55%) who recovered. TSH deficiency was identified in 36 (60%) patients (Fig. 1), with 14 (28%) recovering during medical therapy. Of note, no patient developed new-onset deficit of pituitary hormones during medical treatment. Data on hypopituitarism under medical therapy is summarized in Table 2. Of note, there was no difference in the recovery of deficiencies in the three pituitary axes between patients treated with cabergoline and those receiving bromocriptine (Supplementary Figure).

### Recovery from hypopituitarism: surgery vs. medical treatment

To directly compare hypopituitarism recovery between surgically and medically treated patients, a multivariable logistic regression model was applied. After adjusting for sex, age at treatment initiation, initial tumor size, prolactin normalization, and number of affected pituitary axes, no significant difference was found in the recovery from hypogonadism between the two treatment groups (OR 1.633, 95% CI 0.616–4.4664, *p* = 0.338) (Table 3). Notably, factors influencing hypogonadism recovery were negatively tumor size (OR 0.924, 95% CI 0.883–0.982, *p* = 0.010) and positively prolactin normalization following treatment (OR 5.722, 95% CI 2.067–17.842, *p* = 0.001).

**Table 3:** Parameters affecting recovery of hypogonadism

	OR^1^	95% (CI)^1^	*p*-value
Type of treatment (ref. surgery)	1.63	0.617, 466	0.3
Sex (ref. female)	1.15	0.35, 3.89	0.8
Age at treatment (years)	0.97	0.94, 1.00	0.08
Size of prolactinoma at baseline (mm)	0.92	0.88, 0.98	**< 0.01**
Prolactin normalization (ref. normalization)	5.72	2.07, 17.84	< 0.001
Prevalence of pituitary axis insufficiencies (ref. 2 axes)	0.70	0.24, 2.02	0.5

^1^OR = Odds Ratio, CI = Confidence Interval

Similarly, the type of treatment did not significantly affect the recovery from ACTH deficiency (OR 0.462, 95% CI 0.009–2.012, *p* = 0.319) (Table 4) or TSH deficiency (OR 0.584, 95% CI 0.190–1.768, *p* = 0.339) (Table 5). Both for ACTH deficiency and for TSH deficiency, tumor size was identified as a negative predictor of recovery (Tables 4 and 5). Reflecting this finding, among the giant prolactinomas (those over 4 cm in diameter), 2 out of 12 patients (16%) recovered from hypogonadism, none of the 7 patients recovered from ACTH deficiency, and 1 out of 12 (8%) recovered from TSH deficiency.

**Table 4:** Parameters affecting recovery of ACTH-deficiency

	OR^1^	95% (CI)^1^	*p*-value
Type of treatment (ref. surgery)	0.46	0.01, 2.01	0.3
Sex (ref. female)	1.58	0.23, 11.20	0.6
Age at treatment (years)	1.01	0.97, 1.06	0.4
Size of prolactinoma at baseline (mm)	0.82	0.70, 0.90	< 0.001
Prevalence of pituitary axis insufficiencies (ref. 2 axes)	0.93	0.18, 5.04	0.9

^1^OR = Odds Ratio, CI = Confidence Interval

**Table 5:** Parameters affecting recovery of TSH-deficiency

	OR^1^	95% (CI)^1^	*p*-value
Type of treatment (ref. surgery)	0.58	0.19, 1.76	0.3
Sex (ref. female)	0.59	0.15, 2.41	0.5
Age at treatment (years)	1.02	0.99, 1.06	0.2
Size of prolactinoma at baseline (mm)	0.94	0.89, 0.99	< 0.05
Prevalence of pituitary axis insufficiencies (ref. 2 axes)	1.32	0.39, 4.47	0.7

^1^OR = Odds Ratio, CI = Confidence Interval

## Discussion

In this large European multicenter study, we analyzed the pituitary function of patients with macroprolactinoma and compared for the first time, the hormonal recovery after treatment with dopamine agonists and surgery. Our findings revealed no significant difference between the two treatment modalities in the recovery rates of hypogonadism, ACTH deficiency, and TSH deficiency. A large size of the adenoma and baseline and failure in normalization of prolactin levels, were found to be relevant negative predictors of recovery from hypogonadism. Only a very limited proportion of patients with giant prolactinoma recovered from hypopituitarism.

A prospective study on cabergoline-treated patients showed a similar recovery rate of hypogonadism in about the half of the patients [3]. However, our analysis further demonstrated that recovery of hypogonadism is influenced not only by the degree of prolactin normalization but also by initial adenoma size. This explains why, despite similar hypogonadism prevalence in micro- and macroprolactinomas, recovery is poorer in the latter [7]. The mass effect of macroadenomas, in fact, negatively impacts pituitary function by compressing the gland and disrupting hormone production and release [19].

Recovery of ACTH deficiency is more likely to occur than normalization of other pituitary axes. Our results are in line with one of the largest studies about the topic published so far [9]. In this retrospective analysis of 30 patients with macroprolactinoma, 14 (47%) suffered from ACTH deficiency at baseline, of whom 9 (64%) recovered under medical therapy [9]. In contrast, another study reported that none of their patients recovered from ACTH deficiency during medical therapy [3]. This difference might be explained by the number of patients analyzed (60 and 30 compared to 12 in the latter study). However, it is also possible that the macroprolactinomas in the latter study were larger than those of our patients. Unfortunately, though, Karavitaki et al. [3] did not report the tumor diameters.

We observed that the size of the macroadenomas was a significant predictor not only for the recovery of ACTH but also for TSH deficiency. Published data on the latter is quite variable (with recovery ranging between 0 and 67%) and based on few patients only [3, 5, 20]. We identified that 28% of the patients recovered from TSH deficiency under medical therapy, which is in line with data reported on patients under cabergoline [3]. Confirming the importance of the size of the adenoma in the recovery of hypopituitarism, higher recovery rates of pituitary deficiencies were reported in medically treated patients with macroprolactinomas with smaller diameter [7, 9].

Surgical treatment is an established therapeutic option for macroprolactinomas, and is indicated in cases where medical therapy is unsuccessful or not tolerated. Additionally, as with other pituitary macroadenomas, surgery is essential when addressing neuro-ophthalmologic complications, such as rapid vision loss or cranial nerve paralysis, often resulting from intratumoral hemorrhage or pituitary apoplexy [21]. In our study, prolactin normalization was observed in 60% of cases, consistent with outcomes reported for surgery on suprasellar lesions without visual deficits [11], but higher than remission rates for invasive macroprolactinomas [22]. This difference can most likely be attributed to the extent of cavernous sinus invasion (as higher Knosp grades are associated with a lower likelihood of both complete adenoma removal and prolactin normalization). In our cohort, 22 out of 28 adenomas with postoperative prolactin normalization had a Knosp grade of 0 or 1, while 6 had a Knosp grade of 2. However, given that prolactin normalization significantly impacts hypogonadism recovery, tumor size and its mass effect on the pituitary gland also play a critical role in the recovery of all anterior pituitary axes. Therefore, surgery may be a good option to relieve the pituitary from the pressure exerted by a macroprolactinoma, thereby potentially restoring normal pituitary function. Of note, our results for the post-surgical recovery rates of hypogonadism (42 vs. 45%) and other pituitary axes (which were reported to be 25–35%) are well comparable to the literature [10, 11]. Furthermore, our findings confirm that if an experienced neurosurgeon is involved, the risk of developing new hypopituitarism after surgery is low [22].

Given that 37% of patients underwent surgery due to resistance to medical therapy, one might assume that the surgical group had more aggressive adenomas. However, prolactin levels at diagnosis did not significantly differ between the two groups (801 vs. 1220 µg/L in the surgical and medical groups, respectively; *p* = 0.374), suggesting similar tumor burden [23, 24]. Furthermore, baseline tumor size and pituitary deficits were also comparable, reinforcing the notion that these macroprolactinomas had similar characteristics.

This study highlights the importance of individualized treatment approaches. While surgery may be necessary in case of dopamine agonist resistance or intolerance, medical therapy should be considered in case of extensive cavernous sinus invasion. Given the negative impact of tumor size on recovery, early diagnosis and treatment are crucial for restoring normal pituitary function.

The current study has certainly some limitations. First, this is a retrospective and multicenter nature where the endocrine work-up was not standardized. Second, because of the rarity of the disorder, the number of patients is limited. Third, unfortunately the analysis of growth hormone deficiency was not possible, due to lack of data. Nevertheless, we report the largest cohort of patients with macroprolactinoma with multiple pituitary axes deficiencies and the first direct comparison of surgery and medical therapy for hypopituitarism recovery. Additionally, although the surgeries were performed by three different neurosurgeons, all procedures took place in tertiary pituitary centers with similar expertise and outcomes. Likewise, the patients received medical treatment at tertiary pituitary centers, where endocrinologists had comparable levels of experience.

Of note, an important aspect to consider is that the surgically treated patients were all operated in pituitary tertiary centers, and therefore results on recovery rate or new onset hypopituitarism could be different in not specialized neurosurgical centers.

In conclusion, both surgery and medical treatment offer comparable therapeutic outcomes in patients with macroprolactinoma treated and operated in pituitary tertiary centers. Regardless of the treatment modality applied, recovery of ACTH deficiency is more likely to occur than recovery of other pituitary hormone deficiencies. Considering the importance of tumor size and prolactin normalization for the recovery of pituitary dysfunction, timely and adequate treatment is fundamental.

## Electronic supplementary material

Below is the link to the electronic supplementary material.

Supplementary Material 1
