# Supplementary figures and images for: Recovery of hypopituitarism in macroprolactinomas: a comparison of medical vs. surgical treatment. Results from a European multicenter study
Source: J Endocrinol Invest. 2025 Mar 4;48(6):1363–70. doi: 10.1007/s40618-025-02559-8 (PMC12226669; doi:10.1007/s40618-025-02559-8)

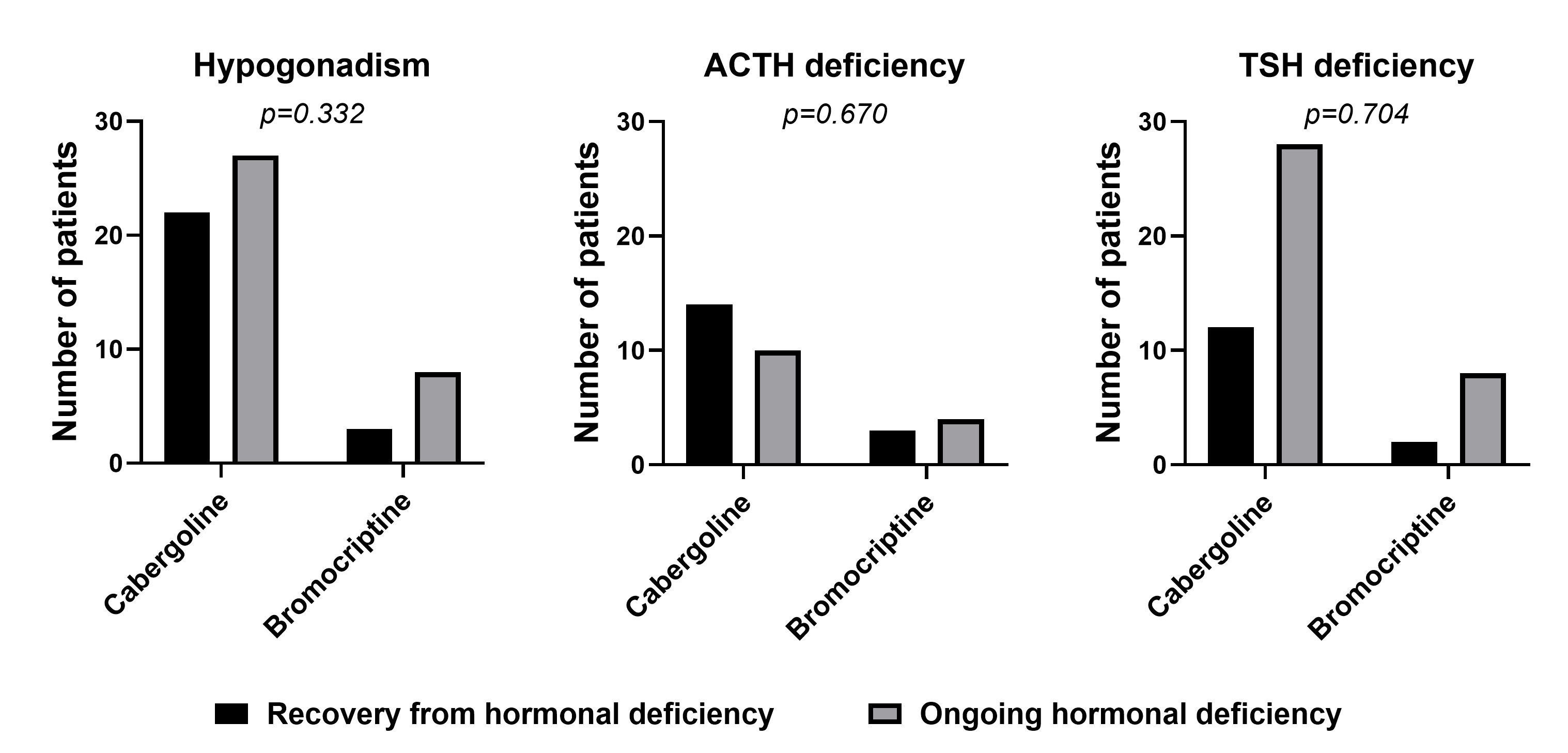

Supplement: Supplementary file 1 — Supplementary Material 1 [file 40618_2025_2559_MOESM1_ESM.tif]
